# Quality of Smartphone Apps Related to Panic Disorder

**DOI:** 10.3389/fpsyt.2015.00096

**Published:** 2015-07-14

**Authors:** Mathias Van Singer, Anne Chatton, Yasser Khazaal

**Affiliations:** ^1^Geneva University, Geneva, Switzerland; ^2^Geneva University Hospitals, Geneva, Switzerland

**Keywords:** Internet, apps, smartphone, panic disorder, anxiety disorder, cognitive behavior therapy

## Abstract

Quality of smartphone apps related to panic: smartphone apps have a growing role in health care. This study assessed the quality of English-language apps for panic disorder (PD) and compared paid and free apps. Keywords related to PD were entered into the Google Play Store search engine. Apps were assessed using the following quality indicators: accountability, interactivity, self-help score (the potential of smartphone apps to help users in daily life), and evidence-based content quality. The Brief DISCERN score and the criteria of the “Health on the Net” label were also used as content quality indicators as well as the number of downloads. Of 247 apps identified, 52 met all inclusion criteria. The content quality and self-help scores of these PD apps were poor. None of the assessed indicators were associated with payment status or number of downloads. Multiple linear regressions showed that the Brief DISCERN score significantly predicted the content quality and self-help scores. Poor content quality and self-help scores of PD smartphone apps highlight the gap between their technological potential and the overall quality of available products.

## Introduction

Panic disorder (PD) is a common anxiety disorder associated with an important social and economic burden (1, 2). Available treatments include pharmacotherapy and cognitive–behavioral therapy (CBT) (3). Such treatments have, however, been insufficiently disseminated in clinical settings (4).

Smartphones are widely used worldwide (5, 6) and have a growing role in health care (7–10). Concerns about the regulation of medical smartphone apps are, at the same time, rising among public administrations and the scientific community (9, 11, 12). The U.S. Food and Drug Administration regulates some health-related apps, as it does medical devices. Smartphone apps related to psychiatric conditions do not yet fall under this regulation (13).

A number of recent studies have assessed the quality of medically oriented apps in various fields, such as smoking cessation, weight management, sleep, cancer, and diabetes (14–36). While acknowledging the potential opportunity offered by apps-related technologies, these studies concluded that the apps available from different stores, with few exceptions, were of overall poor quality. A gap was furthermore found between the considerable number of apps related to medical conditions available in stores and the low number of peer-reviewed papers about them (37). In particular, despite their potential to improve health care, mental health apps currently available in stores lack scientific evidence about their efficacy (38). With few exceptions (39–41), preliminary findings reported for health apps were similar to previous findings on the poor quality of health information websites (42–46).

One may hypothesize that persons with PD could benefit from the development of well-conceived apps. Indeed, Internet-based CBT has previously been shown to offer some efficacy in PD (47–50), and several studies are under way to evaluate apps designed specifically for the treatment of PD (51, 52). Nonetheless, developers have not waited for scientific evidence to create apps for PD, as many are already available on smartphone stores. To our knowledge, no studies have yet been performed to rate these apps.

In the present study, we aimed to assess the quality of English-language PD-related apps available on the Google Play Store like any layperson searching for an app related to PD on the Google Play Store. It is a descriptive and exploratory study of what it is possible to find. Furthermore, the study aimed to compare free and paid apps. We furthermore assessed the factors associated with the main quality indicators, as well as the links between the quality indicators and users’ ratings (star ratings, as reported on the Google Play page) and downloads.

## Materials and Methods

### Selection of apps

A keyword search was performed between February and March 2014 to produce a comprehensive list of PD-related apps that were accessible in English on the Google Play Store. The Google play account was set to English United Kingdom language and linked to a mobile phone, which was registered on a Swiss mobile network.

Google is the developer of the Android operating system, the most widely used smartphone operating system in the world (53). The following queries were entered into the Google Play search engine: “stop panic,” “stop panic attack,” “panic attack,” “PD,” “anxiety attacks,” and “anxiety disorder.”

Studies of Internet users have shown that most people rarely search beyond the first 20 retrieved results (54). However, we extended the coverage of the present study to the first 50 free apps and to the first 30 paid apps for each tag to obtain the most comprehensive list of apps. Apps were included if they were related to PD. Exclusion criteria were as follows: the app could not be downloaded after more than three attempts, the app was not in English, or the app was a book or an article.

### Evaluation of apps

Apps were reviewed on an HTC One Android 4.3. They were assessed by using tools reported in previous studies, tools adapted from quality evaluation studies of websites (55–59), and tools described in other studies on the quality of smartphone apps (15, 16, 23, 30, 31). The assessment instruments are described below.

#### Google Play’s Page and Functionalities of Apps

As reported by other investigators (15, 30), we extracted a number of items from the Google Play page, such as number of downloads and ratings of the apps.

#### Self-help Model

A self-help model assessment tool for PD was used (Table 1). The model was based on the potential of smartphone apps to help users in daily life and on the second edition of the *Practice Guideline for the Treatment of Patients with Panic Disorder* by the American Psychiatric Association (3).

**Table 1:** **The self-help model**.

**General items, quoted as follows**			
Automated feedback	0 = absence of feedback	1 = non-tailored feedback	2 = tailored feedback
Biofeedback	0 = absent	1 = present
Personal statistics	0 = absent	1 = present
Promotion of non-evidence-based features	0 = present	1 = absent
**Items usually present in panic-focused CBT, quoted as follows**
	0 = absent	1 = present, only informative	2 = present with interactive help
Psychoeducation			
Self-monitoring			
Cognitive restructuring			
Exposure to fear cues			
Modification of anxiety-maintaining behaviors			
Relapse prevention			
Feature to help the user get through a panic attack			

#### Content Quality

As in other studies on Internet websites related to mental health disorders (39–42), evidence-based content quality was assessed according to the availability of information related to the following questions that a patient could search for:

1. How do I know whether I have PD?
2. How do I know whether I have PD with or without agoraphobia?
3. Can I estimate the severity of my disease?
4. What are the effective treatments?
5. What are the various useful psychotropic drugs for PD, and what are their side effects?
6. What difficulties might I encounter during or after treatment?
7. What psychotherapies are effective in the treatment of PD?

Answers found on the apps were assessed on the basis of the American Psychiatric Association practice guideline (3). For every request, the coverage (the extent to which the question was addressed) and correctness (the extent to which the answer was right) of the answer were comprehensively scored on a 3-point scale (0 = absent; 1 = partially incorrect or incomplete; 2 = correct and complete). A total content quality score, ranging from 0 to 14, was calculated by combining the scores.

#### Interactivity

Interactivity (Table 2) was measured with an adaptation of the Abbott scale (58). Three items were added to the scale: presence of a gamification module, possibility of personalizing the user’s profile (avatar, color, sound), and tailoring of the app upon use.

**Table 2:** **Assessment scales**.

Scale		Coding
**Interactivity, Abbott’s scale**		**0 = absent; 1 = present**
1	Internal search engine	0 1
2	The presence of audio or video support	0 1
3	Questionnaire of satisfaction	0 1
4	Possibility of sending complaints and requests to the webmasters or to the authors?	0 1
5	Presence of forums, chat, or social networking?	0 1
6	Presence of a “game-like” module?	0 1
7	Tailoring of app	0 1
8	Personalization of app/user’s profile (avatar, color, sound)?	0 1
**HON**		**0 = absent; 1 = present**
1	Authoritative	0 1
2	Complementarity	0 1
3	Privacy	0 1
4	Attribution	0 1
5	Justifiability	0 1
6	Transparency	0 1
7	Financial disclosure	0 1
8	Advertising policy	0 1
**Brief DISCERN**		**1 = no, yes = 5, in between = partially**
1	Is it clear what sources of information were used to compile the publication?	1–5
2	Is it clear when the information used or reported in the publication was produced?	1–5
3	Does it describe how each treatment works?	1–5
4	Does the publication describe the benefits of each treatment?	1–5
5	Does it describe the risks of each treatment?	1–5
6	Does it describe how the treatment choices affect overall quality of life?	1–5
**Silberg scale for accountability**		**0 = absent; 1 = present**
1	Name of the author and qualification	0 1
2	Affiliation	0 1
3	Sources and references	0 1
4	Property of site	0 1
5	Sponsorship	0 1
6	Advertising	0 1
7	Date of creation	0 1
8	Date of last update	0 1
9	Updated within last 6 months	0 1

#### Health on the Net Code

The health on the net code (HON) label (55) was created for websites that focus on ethical standards in online publishing. Usually, a website requests evaluation, after which the label is awarded. In the absence of the common use of this label by the apps, we assessed whether they respected the HON criteria (Table 2).

#### Brief DISCERN

The Brief DISCERN (56) is a six-item (Table 2) assessment tool adapted from the DISCERN instrument (60). It is used as a potential indicator to estimate the quality of the information about the choice of treatment in websites. The Brief DISCERN includes six items on a five-point scale (1 = not at all; 5 = completely). The first two items identify the transparency of information sources; the other four items estimate the quality of the information regarding treatment. A cutoff score of ≥16 has previously been associated with good content quality scores of health-related websites (56).

#### Accountability

Accountability was estimated with the Silberg scale (57), which includes authorship (names of authors, affiliation, and references), attribution (sources and references), disclosure (property of site, sponsorship, and advertising), and currency (date of creation, modification of site, and updating in the last 6 months). A total score ranging from 0 to 9 (1 point for each item if present) was calculated for each app (Table 2).

### Statistical analyses

Statistical analyses were performed with SPSS software (version 18.0, Chicago, IL, USA). An initial exploratory analysis involved the calculation of proportions, as well as means and SDs, of the above-mentioned outcome measures. Next, we compared paid apps with free apps in bivariate analyses by using parametric tests (*t*-test, chi-square, or Fisher’s exact test) or non-parametric tests (median test) when appropriate. Finally, we computed prediction models by using multiple linear regressions for two variables of interest.

Applying the principle of model parsimony and statistical relevance, the selection of the independent variables was driven by the objective to find the simplest model (i.e., contain a small number of variables):

(1) that adequately fits the data and
(2) that explains most of the variance in the dependent variable (the highest value of R2).

Beforehand, the relevance of the independent variables for the prediction of each dependent variable was discussed among the authors. Taking into account multicollinearity and using the «Enter»method, several subsets of these independent variables were regressed and the model with the least number of variables that still explain a percentage of variance in the dependent variable that is comparable to the percentage explained with all the variables in the equation was retained.

On the one hand, the prediction of content quality was fitted by using the Abbot interactivity scale, the Brief DISCERN score, the number of installs, and the link to paid content (yes vs. no) as the independent variables, and by controlling for payment status (free apps vs. paid apps). On the other hand, the prediction of self-help was assessed with the Brief DISCERN score, whether the app was recommended (yes vs. no), the link to paid content (yes vs. no), and the Silberg accountability scale as the independent variables, controlling for payment status (free apps vs. paid apps). For all analyses, a significance level of *p* ≤ 0.05 was used.

Payment status was hypothesized to matter as a potential confounder on the ground that it could moderate the impact of the independent variables on the dependent variable. Using the 10% change rule of thumb, confounding was considered present if the measures of association changed by 10% or more and absent otherwise. Measuring this change implied running the model twice: without and with the confounder.

## Results

**Figure 1:**
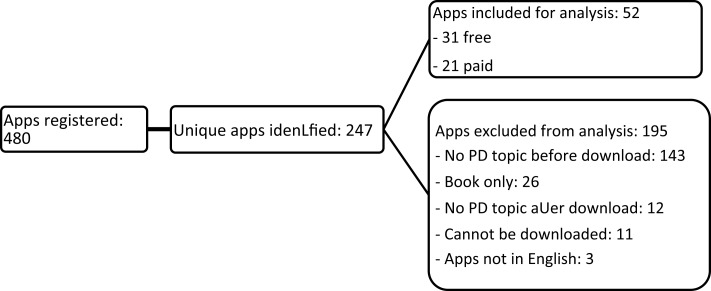
**Study flow chart**. PD, panic disorder.

A total of 480 apps were found (50 free apps and 30 paid apps for each of the six keywords). The search with these keywords highlighted several duplicates among the apps identified. After their removal, of the 247 remaining unique apps, 52 were retained for analysis and 195 were excluded (Figure 1).

One app offered only a self-assessment tool aiming to help user to screen for a possible PD. The other apps are designed to help user manage their symptoms (via information, assessments, and techniques to deal with PD). Most of the apps (58.1% of free apps and 61.9% of paid apps) recommend the user to consult a medical doctor if suffering from symptoms of PD.

There is furthermore a wide variability on the contents of the apps. For example, one of the most downloaded app offers features like psychoeducation, audio’s for relaxation, and mindfullness as well as a diary tool to record panic events. Some apps offer interactive modules to face panic attacks. For example, one app tells the user to control his breathing while giving instruction on the screen with pictograms. Other apps offer discussion forum. Some applications are selling various products, such as books or medicinal herbs.

The characteristics of paid and free unique apps are reported in Table 3.

**Table 3:** **Selected characteristics of smartphone applications (apps) by status (free vs. paid)**.

Characteristics and scores	Free application (*n* = 31)	Paid application (*n* = 21)	*p*-Value
**Play Store and app description**			
Category in Google Play Store			
Book and reference	12.9	0	0.008
Health and fitness	67.7	42.9	0.008
Lifestyle	3.2	33.3	0.008
Medical	16.1	23.8	0.008
Mean rank (SD)	34.6 (10.0)	32.8 (12.5)	0.6
Mean star rating	2.2 (2.0)	2.2 (2.1)	1.0
Number of raters			
None	38.7	47.6	0.3
Between 1 and 10	29.0	38.1	
More than 10	32.3	14.3	
**App recommended?**			
No	29.0	38.1	0.5
Number of downloads			
Less than 100	25.8	71.4	0.005
100–500	29.0	9.5	
More than 500	45.2	19.0	
Identity of the website developer?			
No	38.7	14.3	0.06
Privacy policy?			
No	83.9	85.7	1.0
Clear purpose of app?			
No	12.9	4.8	0.6
Privacy authorization explanation?			
No	90.3	100.0	0.3
Disclaimer?			
No	87.1	100.0	0.1
Linked app?			
No	90.3	90.3	–
Tutorial present?			
No	93.5	90.5	1.0
User profiles?			
No	87.1	90.5	1.0
Password protection?			
No	80.6	90.5	0.4
Backup?			
No	96.8	100.0	1.0
Bug report?			
No	87.1	71.4	0.2
Publicity within the app?			
No	38.7	100.0	<0.0005
Link to paid content from the play store app’s page?			
No	38.7	61.9	0.1
Disclaimer?			
No	80.6	81.0	1.0
Privacy policy?			
No	83.9	85.7	1.0
Help system?			
No	87.1	66.7	0.1
App designed as a “stand-alone”?			
No	71.0	23.8	0.001
App designed to be used with external expert assistance/supervision?			
No	90.3	76.2	0.2
Advice to consult doctor?			
No	41.9	38.1	0.8
Content conformed to description in Google Play Store?			
No	6.5	4.8	1.0
**Self-help scores (0-21)**	3.9 (3.1)	3.1 (2.7)	0.4
**Content quality scores (0-14)**	2.5 (3.0)	1.2 (2.5)	0.1
**Abbott interactivity scale (0-8)**	1.6 (1.5)	1.7 (1.1)	0.9
**HON**			
0–2 criteria filled	80.6	38.1	0.002
3–7 criteria filled	19.4	61.7	
All 8 criteria filled	0	0	
**Brief DISCERN**			
< 16	64.5	85.7	0.1
≥ 16	35.3	14.3	
**Silberg accountability scale (0-9)**	2.4 (1.7)	2.6 (1.3)	0.6

*Summary statistics report variable mean and standard deviation (SD), or percentage, as appropriate*.

The two subgroups, paid and free apps, were similar, although we found several differences. For instance, the variable publicity appeared to be present in 61.3% of the free apps group but in none of the paid apps group, with a *p*-value of <0.0005. In addition, the HON criteria were more fulfilled in the paid apps group than in the free apps group (*p*-value 0.002). As shown in Table 3, the overall adherence to HON criteria was low: none of the assessed apps fulfilled the eight criteria. Significantly more apps that were designed to be stand-alone (no additional app content) were found in the paid apps group than in the free apps group (*p*-value 0.001). Both paid and free apps groups had low content quality and low self-help scores, with no differences between the two groups.

### Regression results

Content quality was regressed on a set of independent variables, namely the Abbot interactivity scale, the Brief DISCERN score, the number of downloads, the link to paid content controlling for payment status. We found that the Abbot interactivity scale and the Brief DISCERN score significantly predicted the content quality (*p* = 0.01 and *p* < 0.0005, respectively), but not the number of downloads (*p* = 0.9), or the link to paid content (*p* = 0.1). After careful examination of the regression coefficients without and with payment status, no confounding effect could be imputed to this variable. The full model performed well with an adjusted *R*^2^ of about 70%.

Another regression model predicted the self-help score with the Silberg accountability scale, the Brief DISCERN score, whether the app was recommended, and the link to paid content as the independent variables, controlling for payment status. This model performed less well than the preceding model, as shown by an adjusted *R*^2^ of 54.4%. We found that the Silberg accountability scale (*p* < 0.0005) and the Brief DISCERN score (*p* = 0.03) significantly predicted the self-help score, but not whether the app was recommended (*p* = 0.4), or the link to paid content (*p* = 0.5). After due consideration of the regression coefficients without and with payment status, we did not detect a confounding effect of this variable.

## Discussion

In this study, we aimed to assess the quality of English-language smartphone apps for PD. In particular, we evaluated the content quality and self-help scores with instruments that were adapted from other studies on the content quality of medical websites and from previous assessment studies on medical smartphone apps.

In consideration of the lack of specific studies with similar purpose to our study, and of the potentially high interactivity of apps in daily life, we adapted some assessment tools for the study herein. Abbott’s scale of interactivity (58) was adapted to match the specificity of smartphone apps, as described in the Section “Materials and Methods.” Another important adaptation was the self-help tool. It may be a helpful indicator for apps assessment and development, especially for those based on CBT treatment models. The low scores obtained with the tool may underline the gap between the clinical potential offered by apps technology and its rather low level of clinical development. The self-help model was based on CBT for two reasons: first, its easy translation into eHealth, as shown by important developments related to Internet-based therapy for mental health disorders (48, 61–64); second, its validity for the treatment of PD (3). The self-help model score is probably also useful for other CBT treatment apps. Further studies on apps for mental health and about the instruments proposed here are warranted.

Similar to the results reported in other studies on health-related apps, the mean content quality (14–16, 19, 23, 30, 31, 34, 35) and self-help scores were low in our study. Most apps were insufficiently evidence based; furthermore, the technological capabilities were underused in most of the available PD-related apps. As shown in website studies (40, 41, 56), the Brief DISCERN score (56) is linked to content quality scores, as well as to the self-help score specifically developed for CBT-based app assessment. In the present study, measures such as accountability and interactivity were associated with the main quality indicators, such as content quality and self-help scores, as was previously found in some (39, 41, 65), but not all (66), studies on health-related websites.

Factors related to the community success of a given app, such as the number of downloads and whether the app was recommended, as well as factors linked to the economic model, such as payment status or a link to paid content, were not associated with content quality or self-help scores. This is somewhat surprising, particularly in regard to the number of downloads. One might expect better quality for the most downloaded apps. The results are possibly limited by the assessments of apps found only on the Google Play Store as well as by the small number of apps with a high amount of downloads (only three apps with more than 5000 downloads).

The number of active users (unavailable on the Google Play Store) would, however, probably be more informative for the sustained success of a given app after the initial download.

Payment status was not associated with the quality indicators assessed. Further studies may assess in more details the commercial strategy linked to the development model related to health-related apps.

The link found between payment status and publicity reflects some differences in the commercial model. Other aspects should, however, be included in further assessments (i.e., marketing strategies, interaction with users.).

Our study contains several limitations. We assessed only apps from the Google Play Store and not the Apple Appstore or others. This aspect limits the generalization of the study findings.

In addition, the keywords for the search used in this study might be different from those used by people with PD, and we may have missed PD-related apps on the Google Play Store. Furthermore, the results may differ depending on the country and the language setting of the Google Play store.

Nonetheless, the study suggests possible modifications to medical eHealth assessments through the proposal of adaptations to apps. Despite expectations about the potential of PD apps to improve treatments (51, 52), the apps available to users from stores to date need to be improved and to include more patterns of evidence-based information, more interactive assessments, such as ecological momentary assessments (67), and more self-help options.

## Conflict of Interest Statement

The authors declare that the research was conducted in the absence of any commercial or financial relationships that could be construed as a potential conflict of interest.

## References

[1] Kessler RC Chiu WT Jin R Ruscio AM Shear K Walters EE. The epidemiology of panic attacks, panic disorder, and agoraphobia in the National Comorbidity Survey Replication. Arch Gen Psychiatry (2006) 63(4):415–24. 10.1001/archpsyc.63.4.415 16585471 PMC1958997

[2] Greenberg PE Sisitsky T Kessler RC Finkelstein SN Berndt ER Davidson JR The economic burden of anxiety disorders in the 1990s. J Clin Psychiatry (1999) 60(7):427–35. 10.4088/JCP.v60n0702 10453795

[3] Association AP Practice Guideline for the Treatment of Patients with Panic Disorder. Second edition ed Washington, DC: American Psychiatric Association (2009).

[4] Schubert JR Coles ME Heimberg RG Weiss BD. Disseminating treatment for anxiety disorders step 2: peer recommendations to seek help. J Anxiety Disord (2014) 28(7):712–6. 10.1016/j.janxdis.2014.07.010 25145571 PMC4160353

[5] Dubai WD. Smartphone Users Around the World – Statistics and Facts. (2014). Available from: http://www.go-gulf.com/blog/smartphone/

[6] eMarketer. Smartphone Users Worldwide will Total 1.75 Billion in 2014. (2014). Available from: http://www.emarketer.com/Article/Smartphone-Users-Worldwide-Will-Total-175-Billion-2014/1010536

[7] Mosa AS Yoo I Sheets L. A systematic review of healthcare applications for smartphones. BMC Med Inform Decis Mak (2012) 12:67. 10.1186/1472-6947-12-67 22781312 PMC3534499

[8] Proudfoot J Parker G Hadzi Pavlovic D Manicavasagar V Adler E Whitton A. Community attitudes to the appropriation of mobile phones for monitoring and managing depression, anxiety, and stress. J Med Internet Res (2010) 12(5):e64. 10.2196/jmir.1475 21169174 PMC3057321

[9] Cortez NG Cohen IG Kesselheim AS. FDA regulation of mobile health technologies. N Engl J Med (2014) 371(4):372–9. 10.1056/NEJMhle1403384 25054722

[10] Codyre P. Will an app fill the gap? Innovative technology to provide point-of-care information. Front Public Health (2014) 2:9. 10.3389/fpubh.2014.00009 24551835 PMC3914210

[11] Becker S Miron-Shatz T Schumacher N Krocza J Diamantidis C Albrecht UV. mHealth 2.0: experiences, possibilities, and perspectives. JMIR mHealth uHealth (2014) 2(2):e24. 10.2196/mhealth.3328 25099752 PMC4114478

[12] Boulos MN Brewer AC Karimkhani C Buller DB Dellavalle RP. Mobile medical and health apps: state of the art, concerns, regulatory control and certification. Online J Public Health Inform (2014) 5(3):229. 10.5210/ojphi.v5i3.4814 24683442 PMC3959919

[13] FDA. FDA issues Final Guidance on Mobile Medical Apps. (2013). Available from: http://www.fda.gov/NewsEvents/Newsroom/PressAnnouncements/ucm369431.htm 24358515

[14] Abroms LC Lee Westmaas J Bontemps-Jones J Ramani R Mellerson J. A content analysis of popular smartphone apps for smoking cessation. Am J Prev Med (2013) 45(6):732–6. 10.1016/j.amepre.2013.07.008 24237915 PMC3836190

[15] Abroms LC Padmanabhan N Thaweethai L Phillips T. iPhone apps for smoking cessation: a content analysis. Am J Prev Med (2011) 40(3):279–85. 10.1016/j.amepre.2010.10.032 21335258 PMC3395318

[16] Breton ER Fuemmeler BF Abroms LC. Weight loss-there is an app for that! but does it adhere to evidence-informed practices? Transl Behav Med (2011) 1(4):523–9. 10.1007/s13142-011-0076-5 24073074 PMC3717669

[17] Cohn AM Hunter-Reel D Hagman BT Mitchell J. Promoting behavior change from alcohol use through mobile technology: the future of ecological momentary assessment. Alcohol Clin Exp Res (2011) 35(12):2209–15. 10.1111/j.1530-0277.2011.01571.x 21689119 PMC3221771

[18] Choi J Noh GY Park DJ. Smoking cessation apps for smartphones: content analysis with the self-determination theory. J Med Internet Res (2014) 16(2):e44. 10.2196/jmir.3061 24521881 PMC3936270

[19] Pagoto S Schneider K Jojic M DeBiasse M Mann D. Evidence-based strategies in weight-loss mobile apps. Am J Prev Med (2013) 45(5):576–82. 10.1016/j.amepre.2013.04.025 24139770

[20] Bennett ME Toffey K Dickerson F Himelhoch S Katsafanas E Savage CLA Review of android apps for smoking cessation. J Smok Cessat (2014). 10.1017/jsc.2014.1

[21] Schoffman DE Turner-McGrievy G Jones SJ Wilcox S. Mobile apps for pediatric obesity prevention and treatment, healthy eating, and physical activity promotion: just fun and games? Transl Behav Med (2013) 3(3):320–5. 10.1007/s13142-013-0206-3 24073184 PMC3771006

[22] Direito A Dale LP Shields E Dobson R Whittaker R Maddison R. Do physical activity and dietary smartphone applications incorporate evidence-based behaviour change techniques? BMC Public Health (2014) 14:646. 10.1186/1471-2458-14-646 24965805 PMC4080693

[23] West JH Hall PC Hanson CL Barnes MD Giraud-Carrier C Barrett J. There’s an app for that: content analysis of paid health and fitness apps. J Med Internet Res (2012) 14(3):e72. 10.2196/jmir.1977 22584372 PMC3799565

[24] Azar KM Lesser LI Laing BY Stephens J Aurora MS Burke LE Mobile applications for weight management: theory-based content analysis. Am J Prev Med (2013) 45(5):583–9. 10.1016/j.amepre.2013.07.005 24139771

[25] Conroy MB Yang K Elci OU Gabriel KP Styn MA Wang J Physical activity self-monitoring and weight loss: 6-month results of the SMART trial. Med Sci Sports Exerc (2011) 43(8):1568–74. 10.1249/MSS.0b013e31820b9395 21200337 PMC4266405

[26] Conroy DE Yang CH Maher JP. Behavior change techniques in top-ranked mobile apps for physical activity. Am J Prev Med (2014) 46(6):649–52. 10.1016/j.amepre.2014.01.010 24842742

[27] Pandey A Hasan S Dubey D Sarangi S. Smartphone apps as a source of cancer information: changing trends in health information-seeking behavior. J Cancer Educ (2013) 28(1):138–42. 10.1007/s13187-012-0446-9 23275239

[28] Breland JY Yeh VM Yu J. Adherence to evidence-based guidelines among diabetes self-management apps. Transl Behav Med (2013) 3(3):277–86. 10.1007/s13142-013-0205-4 24073179 PMC3771007

[29] Demidowich AP Lu K Tamler R Bloomgarden Z. An evaluation of diabetes self-management applications for android smartphones. J Telemed Telecare (2012) 18(4):235–8. 10.1258/jtt.2012.111002 22604278

[30] Huckvale K Car M Morrison C Car J. Apps for asthma self-management: a systematic assessment of content and tools. BMC Med (2012) 10:144. 10.1186/1741-7015-10-144 23171675 PMC3523082

[31] Eng DS Lee JM. The promise and peril of mobile health applications for diabetes and endocrinology. Pediatr Diabetes (2013) 14(4):231–8. 10.1111/pedi.12034 23627878 PMC3837694

[32] Robustillo Cortes Mde L Cantudo Cuenca MR Morillo Verdugo R Calvo Cidoncha E. High quantity but limited quality in healthcare applications intended for HIV-infected patients. Telemed J E Health (2014) 20(8):729–35. 10.1089/tmj.2013.0262 24849001

[33] Mobasheri MH Johnston M King D Leff D Thiruchelvam P Darzi A. Smartphone breast applications – what’s the evidence? Breast (2014) 23(5):683–9. 10.1016/j.breast.2014.07.006 25153432

[34] Reynoldson C Stones C Allsop M Gardner P Bennett MI Closs SJ Assessing the quality and usability of smartphone apps for pain self-management. Pain Med (2014) 15(6):898–909. 10.1111/pme.12327 24422990

[35] Pandher PS Bhullar KK. Smartphone applications for seizure management. Health Informatics J (2014). 10.1177/1460458214540906 25038202

[36] Behar J Roebuck A Domingos JS Gederi E Clifford GD. A review of current sleep screening applications for smartphones. Physiol Meas (2013) 34(7):R29–46. 10.1088/0967-3334/34/7/R29 23771214

[37] Martinez-Perez B de la Torre-Diez I Lopez-Coronado M. Mobile health applications for the most prevalent conditions by the World Health Organization: review and analysis. J Med Internet Res (2013) 15(6):e120. 10.2196/jmir.2600 23770578 PMC3713954

[38] Donker T Petrie K Proudfoot J Clarke J Birch MR Christensen H. Smartphones for smarter delivery of mental health programs: a systematic review. J Med Internet Res (2013) 15(11):e247. 10.2196/jmir.2791 24240579 PMC3841358

[39] Morel V Chatton A Cochand S Zullino D Khazaal Y. Quality of web-based information on bipolar disorder. J Affect Disord (2008) 110(3):265–9. 10.1016/j.jad.2008.01.007 18280578

[40] Zermatten A Khazaal Y Coquard O Chatton A Bondolfi G. Quality of web-based information on depression. Depress Anxiety (2010) 27(9):852–8. 10.1002/da.20665 20099271

[41] Klila H Chatton A Zermatten A Khan R Preisig M Khazaal Y. Quality of web-based information on obsessive compulsive disorder. Neuropsychiatr Dis Treat (2013) 9:1717–23. 10.2147/NDT.S49645 24235835 PMC3821751

[42] Khazaal Y Fernandez S Cochand S Reboh I Zullino D. Quality of web-based information on social phobia: a cross-sectional study. Depress Anxiety (2008) 25(5):461–5. 10.1002/da.20381 17960640

[43] Khazaal Y Chatton A Cochand S Jermann F Osiek C Bondolfi G Quality of web-based information on pathological gambling. J Gambl Stud (2008) 24(3):357–66. 10.1007/s10899-008-9095-7 18373182

[44] Khazaal Y Chatton A Zullino D Khan R. HON label and DISCERN as content quality indicators of health-related websites. Psychiatr Q (2012) 83(1):15–27. 10.1007/s11126-011-9179-x 21547515

[45] Eysenbach G Powell J Kuss O Sa ER. Empirical studies assessing the quality of health information for consumers on the world wide web: a systematic review. JAMA (2002) 287(20):2691–700. 10.1001/jama.287.20.2691 12020305

[46] Chan DS Willicombe A Reid TD Beaton C Arnold D Ward J Relative quality of internet-derived gastrointestinal cancer information. J Cancer Educ (2012) 27(4):676–9. 10.1007/s13187-012-0408-2 22918796

[47] Shandley K Austin DW Klein B Pier C Schattner P Pierce D Therapist-assisted, internet-based treatment for panic disorder: can general practitioners achieve comparable patient outcomes to psychologists? J Med Internet Res (2008) 10(2):e14. 10.2196/jmir.1033 18487138 PMC2483919

[48] Carlbring P Nilsson-Ihrfelt E Waara J Kollenstam C Buhrman M Kaldo V Treatment of panic disorder: live therapy vs. self-help via the internet. Behav Res Ther (2005) 43(10):1321–33. 10.1016/j.brat.2004.10.002 16086983

[49] Carlbring P Andersson G Kaldo V. State-of-the-art treatment via the internet: an optimistic vision of the future. Cogn Behav Ther (2011) 40(2):79–81. 10.1080/16506073.2011.575591 25155811

[50] Hadjistavropoulos HD Pugh NE Nugent MM Hesser H Andersson G Ivanov M Therapist-assisted internet-delivered cognitive behavior therapy for depression and anxiety: translating evidence into clinical practice. J Anxiety Disord (2014) 28(8):884–93. 10.1016/j.janxdis.2014.09.018 25445078

[51] Lindner P Ivanova E Ly KH Andersson G Carlbring P. Guided and unguided CBT for social anxiety disorder and/or panic disorder via the internet and a smartphone application: study protocol for a randomised controlled trial. Trials (2013) 14:437. 10.1186/1745-6215-14-437 24351088 PMC3878326

[52] Ebenfeld L Stegemann SK Lehr D Ebert DD Jazaieri H Van Ballegooijen W Efficacy of a hybrid online training for panic symptoms and agoraphobia: study protocol for a randomized controlled trial. Trials (2014) 15(1):427. 10.1186/1745-6215-15-427 25370504 PMC4233107

[53] McCracken H Who’s winning, iOS or Android? all the numbers, all in one place. Time. (2013). Available from: http://techland.time.com/2013/04/16/ios-vs-android/

[54] Eysenbach G Kohler C. How do consumers search for and appraise health information on the world wide web? qualitative study using focus groups, usability tests, and in-depth interviews. BMJ (2002) 324(7337):573–7. 10.1136/bmj.324.7337.573 11884321 PMC78994

[55] Boyer C Selby M Scherrer JR Appel RD. The health on the net code of conduct for medical and health websites. Comput Biol Med (1998) 28(5):603–10. 10.1016/S0010-4825(98)00037-7 9861515

[56] Khazaal Y Chatton A Cochand S Coquard O Fernandez S Khan R Brief DISCERN, six questions for the evaluation of evidence-based content of health-related websites. Patient Educ Couns (2009) 77(1):33–7. 10.1016/j.pec.2009.02.016 19372023

[57] Silberg WM Lundberg GD Musacchio RA. Assessing, controlling, and assuring the quality of medical information on the internet: caveant lector et viewor – let the reader and viewer beware. JAMA (1997) 277(15):1244–5. 10.1001/jama.1997.03540390074039 9103351

[58] Abbott VP. Web page quality: can we measure it and what do we find? A report of exploratory findings. J Public Health Med (2000) 22(2):191–7. 10.1093/pubmed/22.2.191 10912558

[59] eEurope. Quality criteria for health related websites. J Med Internet Res (2002) 4(3):E15. 10.2196/jmir.4.3.e15 12554546 PMC1761945

[60] Charnock D Shepperd S. Learning to DISCERN online: applying an appraisal tool to health websites in a workshop setting. Health Educ Res (2004) 19(4):440–6. 10.1093/her/cyg046 15155597

[61] Andersson G. Using the internet to provide cognitive behaviour therapy. Behav Res Ther (2009) 47(3):175–80. 10.1016/j.brat.2009.01.010 19230862

[62] Andersson G Cuijpers P Carlbring P Riper H Hedman E. Guided Internet-based vs. face-to-face cognitive behavior therapy for psychiatric and somatic disorders: a systematic review and meta-analysis. World Psychiatry (2014) 13(3):288–95. 10.1002/wps.20151 25273302 PMC4219070

[63] Cuijpers P van Straten A Andersson G. Internet-administered cognitive behavior therapy for health problems: a systematic review. J Behav Med (2008) 31(2):169–77. 10.1007/s10865-007-9144-1 18165893 PMC2346512

[64] van der Spek N Vos J van Uden-Kraan CF Breitbart W Cuijpers P Knipscheer-Kuipers K Effectiveness and cost-effectiveness of meaning-centered group psychotherapy in cancer survivors: protocol of a randomized controlled trial. BMC Psychiatry (2014) 14(1):22. 10.1186/1471-244X-14-22 24467861 PMC3942178

[65] Khazaal Y Chatton A Cochand S Zullino D. Quality of web-based information on cocaine addiction. Patient Educ Couns (2008) 72(2):336–41. 10.1016/j.pec.2008.03.002 18423952

[66] Griffiths KM Christensen H. Quality of web based information on treatment of depression: cross sectional survey. BMJ (2000) 321(7275):1511–5. 10.1136/bmj.321.7275.1511 11118181 PMC27555

[67] Walz LC Nauta MH Aan Het Rot M. Experience sampling and ecological momentary assessment for studying the daily lives of patients with anxiety disorders: a systematic review. J Anxiety Disord (2014) 28(8):925–37. 10.1016/j.janxdis.2014.09.022 25445083

